# Frequencies of thyroid-related diagnoses and dyslipidemia among Japanese women with menstrual-related disorders aged under 40 years: A nationwide cross-sectional study using health insurance claims data

**DOI:** 10.1371/journal.pone.0361575

**Published:** 2026-10-30

**Authors:** Tatsuya Yoshihara, Yosuke Ono, Harumasa Arita, Akiko Nakagomi, Dai Miyashita, Kota Tanaka, Satoko Sasatsu, Osamu Yoshino

**Affiliations:** 1 Department of Obstetrics and Gynecology, University of Yamanashi, Yamanashi, Japan; 2 Rohto Pharmaceutical Co., Ltd., Osaka, Japan; Medical Park Minatomirai, JAPAN

## Abstract

Women with endometriosis and dysmenorrhea are increasingly recognized as having systemic comorbidities beyond pelvic disease, but large-scale data on endocrine and metabolic comorbidities across a broad menstrual-related disorders population remain limited. Using the Japan Medical Data Center Claims Database, we conducted a nationwide cross-sectional study of women aged under 40 years enrolled in 2023. Women with confirmed diagnoses of endometriosis (ICD-10: N80) and/or dysmenorrhea (N94.4 or N94.6) were classified as the menstrual-related disorders group (n = 257,897); those without such diagnoses were the control group (n = 3,183,032). The primary outcomes were the frequencies of thyroid-related diagnoses (hypothyroidism [E03], non-toxic goiter [E04], hyperthyroidism [E05], thyroiditis [E06], and other thyroid disorders [E07]) and dyslipidemia (E78). Stratified analyses were performed according to prescription for low-dose estrogen–progestin preparations (LEP) or dienogest (DNG). All thyroid-related diagnoses were recorded more frequently among women with menstrual-related disorders than among controls. Hypothyroidism was identified in 2.1% of women with menstrual-related disorders versus 0.8% of controls (prevalence ratio [PR] 2.46; 95% CI 2.39–2.54); the corresponding PR was 3.11 (95% CI 2.99–3.24) in the endometriosis subgroup (n = 96,863). Dyslipidemia was recorded in 5.2% of women with menstrual-related disorders versus 1.4% of controls (PR 3.73; 95% CI 3.66–3.80; prevalence difference +3.78 percentage points). The endometriosis subgroup consistently showed the highest frequencies. These associations remained statistically significant after adjustment for age using the Mantel-Haenszel method (adjusted PR 1.45–3.02 across all comparisons), although adjusted estimates were consistently lower than crude estimates. Among women with menstrual-related disorders, hypothyroidism was more frequent in those without LEP/DNG prescriptions (3.4%) than in those with prescriptions (1.5%), whereas dyslipidemia frequencies were similar (5.2% vs. 5.1%). These findings indicate that young Japanese women with menstrual-related disorders have substantially higher frequencies of thyroid-related diagnoses and dyslipidemia than controls, particularly those with endometriosis. Surveillance bias, age confounding, and confounding by indication cannot be excluded because of the cross-sectional claims-based design.

## Introduction

Menstrual-related disorders, including dysmenorrhea and endometriosis, are major health problems among women of reproductive age and are closely associated with chronic pelvic pain, infertility, and impaired quality of life [1–3]. Dysmenorrhea is a common gynecological symptom among young women and can affect school attendance, work productivity, and daily activities [4–6]. Endometriosis is associated with dysmenorrhea, dyspareunia, chronic pelvic pain, and infertility, and delays in diagnosis and treatment remain an important clinical issue [1,7,8].

Endometriosis has traditionally been regarded as a gynecological disease confined to the pelvis [1]. However, it is increasingly recognized as a systemic disorder involving chronic inflammation, immune dysregulation, oxidative stress, and endocrine alterations [1,9,10]. Women with endometriosis have been reported to have higher frequencies of various comorbidities, including endocrine disorders, metabolic disorders, autoimmune diseases, inflammatory bowel disease, irritable bowel syndrome, psychiatric disorders, and cardiovascular disease [11–14].

Dysmenorrhea is one of the most common gynecological conditions among women of reproductive age, with reported prevalence ranging widely from approximately 16% to 91% depending on the population and diagnostic criteria used, and is particularly common among adolescents and young women [3,4]. Dysmenorrhea has a substantial impact on quality of life, school attendance, and work productivity [5]. In Japanese women, dysmenorrhea is a common menstrual complaint encountered in clinical practice, and its associated symptoms and productivity loss have also been reported [6]. Although dysmenorrhea is often regarded as a self-limiting symptom, recent studies have reported an association between menstrual disorders, including dysmenorrhea, and long-term cardiovascular disease risk, suggesting that dysmenorrhea may serve as a marker of broader cardiovascular and metabolic vulnerability rather than a purely localized gynecological symptom [15]. Despite this growing recognition, most prior studies examining systemic comorbidities in relation to menstrual-related disorders have focused on endometriosis, and comparatively little large-scale evidence exists regarding endocrine and metabolic comorbidities among women with dysmenorrhea, including those without a confirmed diagnosis of endometriosis.

Among these comorbidities, thyroid diseases are important endocrine disorders that are common in women and may be related to menstrual cyclicity, ovulation, fertility, and pregnancy outcomes [16,17]. Dyslipidemia is also an important metabolic disorder associated with long-term cardiovascular risk [18,19]. Although associations of endometriosis with thyroid autoimmunity, thyroid dysfunction, and abnormal lipid profiles have been reported, most previous studies have focused on endometriosis alone [20–22]. Large-scale studies simultaneously evaluating thyroid-related diagnoses and dyslipidemia in a broader population of women with menstrual-related disorders, including dysmenorrhea, remain limited. In addition, real-world evidence regarding the association between prescriptions for low-dose estrogen–progestin preparations (LEP, also known as combined oral contraceptives) or dienogest (DNG) and the frequencies of these diagnoses is scarce.

Therefore, using the Japan Medical Data Center (JMDC) Claims Database, we investigated the association of menstrual-related disorders with the frequencies of thyroid-related diagnoses and dyslipidemia among Japanese women aged <40 years. We also performed subgroup analyses according to the diagnosis of endometriosis or dysmenorrhea and stratified analyses according to prescriptions for LEP or DNG to describe the characteristics of endocrine and metabolic comorbidities among young women with menstrual-related disorders.

## Methods

### Study design and data source

This was a nationwide cross-sectional study using health insurance claims data. We used the JMDC Claims Database, a large-scale Japanese health insurance claims database that includes employees and their dependents covered by employer-based health insurance societies in Japan. The database contains information on outpatient and inpatient diagnoses, medical procedures, and prescriptions. Diagnoses are recorded using International Classification of Diseases, 10th Revision (ICD-10) codes, and prescription information can be extracted using drug codes. The anonymized claims data were accessed for research purposes between August 1, 2025, and September 30, 2025. The data were provided in a fully anonymized format, and the researchers had no access to information that could identify individual participants during or after data acquisition. This study evaluated co-existing recorded diagnoses within the same observation period and was not designed to assess temporal or causal relationships.

### Study population

The study population consisted of women aged <40 years who were enrolled in the JMDC database at any point between January and December 2023. Age was defined as of January 1, 2023.

Women with at least one confirmed diagnosis of a menstrual-related disorder during the observation period were classified into the menstrual-related disorders group. Menstrual-related disorders were defined as endometriosis (ICD-10 code N80) and/or dysmenorrhea (ICD-10 code N94.4 or N94.6). Only diagnoses flagged as confirmed were included in this study. The diagnoses flagged as suspected were excluded to minimize the inclusion of women without an established diagnosis. Women with endometriosis and those with dysmenorrhea were further analyzed as diagnostic subgroups. These subgroups were not mutually exclusive; women with both diagnoses were included in both subgroups. Women without any diagnosis of menstrual-related disorders were classified as the control group.

### Outcomes

The primary outcomes were the frequencies of thyroid-related diagnoses and dyslipidemia during 2023. Thyroid-related diagnoses were defined as hypothyroidism (E03), non-toxic goiter (E04), thyrotoxicosis/hyperthyroidism (E05), thyroiditis (E06), and other thyroid disorders (E07). Dyslipidemia was defined using ICD-10 code E78.

### Hormonal therapy

Among women with menstrual-related disorders, those who received prescriptions for LEP or DNG during the observation period were classified as the LEP/DNG prescription group. LEP included low-dose and ultra-low-dose estrogen–progestin preparations. DNG included dienogest preparations prescribed for dysmenorrhea or endometriosis. Women without prescriptions for either LEP or DNG during the observation period were classified as the non-prescription group.

### Statistical analysis

All statistical analyses were performed using Python version 3.12.0. The frequency of each diagnosis was calculated as the proportion of women with the diagnosis in each group. Using the control group as the reference, prevalence ratios (PR), prevalence differences (PD), and 95% confidence intervals (CI) were calculated for each outcome in the menstrual-related disorders group, endometriosis subgroup, and dysmenorrhea subgroup. The 95% CI for PR were calculated using the log-transformation method. The 95% CI for PD were calculated using the standard error for the difference between two proportions.

To address potential age confounding, we obtained age-stratified tabulations (six categories: < 15, 15–19, 20–24, 25–29, 30–34, and 35–39 years) for each disease outcome and exposure group. Age-adjusted prevalence ratios were calculated using the Mantel-Haenszel method for risk ratios (Greenland–Robins estimator), with 95% confidence intervals estimated using Sato’s variance formula.

To descriptively explore the possibility of surveillance bias, we additionally obtained the distribution of annual outpatient visit frequency among women who received each disease diagnosis, stratified by exposure group (menstrual-related disorders, endometriosis, dysmenorrhea, and controls) and by LEP/DNG prescription status (prescription vs. non-prescription) among women with menstrual-related disorders. Because visit-frequency data were not available for women without each diagnosis, a formal stratified adjustment for healthcare utilization could not be performed. Instead, we compared the mean, standard deviation, median, and interquartile range of outpatient visit frequency between each exposure group and the control group, and separately between the LEP/DNG prescription and non-prescription groups, among women who received the same diagnosis.

### Ethics approval

This study used anonymized claims data provided by JMDC Inc. (Tokyo, Japan). All data used in this study were fully anonymized, and the authors had no access to any personally identifiable information during or after data acquisition. In accordance with the Ethical Guidelines for Medical and Biological Research Involving Human Subjects in Japan, studies using fully anonymized secondary data are exempt from institutional ethics review and informed consent. Therefore, ethics committee approval and individual informed consent were not required. This policy was confirmed by the Institutional Review Board of the University of Yamanashi.

## Results

### Study population

A total of 3,440,929 women aged <40 years who were enrolled in the JMDC Claims Database in 2023 were included in this study. Among them, 257,897 women had confirmed diagnoses of menstrual-related disorders. In the diagnostic subgroups, 96,863 women had endometriosis and 237,727 women had dysmenorrhea. The control group consisted of 3,183,032 women without menstrual-related disorders. During the observation period, 177,366 women received prescriptions for LEP or DNG, accounting for 68.8% of women with menstrual-related disorders.

### Frequency of thyroid-related diagnoses

The frequencies of thyroid-related diagnoses are shown in Table 1 and Fig 1. Comparisons for the endometriosis and dysmenorrhea subgroups are shown in S1 and S2 Tables. Thyroid-related diagnoses were more frequent among women with menstrual-related disorders than among controls.

**Table 1:** Frequency of thyroid-related diagnoses: Menstrual-related disorders vs. controls.

Thyroid-related diagnosis	Menstrual-related disordersn = 257,897	Controlsn = 3,183,032	PR(95% CI)	PD, percentage points(95% CI)
Hypothyroidism (E03)	5,322 (2.1)	26,668 (0.8)	2.46 (2.39 - 2.54)	+1.23 (+1.17 to +1.28)
Non-toxic goiter (E04)	3,485 (1.4)	19,360 (0.6)	2.22 (2.14 - 2.30)	+0.74 (+0.70 to +0.79)
Hyperthyroidism (E05)	4,108 (1.6)	21,846 (0.7)	2.32 (2.25 - 2.40)	+0.91 (+0.86 to +0.96)
Thyroiditis (E06)	3,220 (1.2)	17,922 (0.6)	2.22 (2.14 - 2.30)	+0.69 (+0.64 to +0.73)
Other thyroid disorders (E07)	1,622 (0.6)	5,021 (0.2)	3.99 (3.77 - 4.22)	+0.47 (+0.44 to +0.50)

Values are presented as n (%), prevalence ratios, and prevalence differences. The control group was used as the reference. Thyroid-related diagnosis codes may overlap within the same individual; therefore, the sum of E03–E07 codes does not represent the individual-level prevalence of any thyroid disease after duplicate diagnoses are removed.

PR, prevalence ratio; PD, prevalence difference; CI, confidence interval.

**Fig 1:**
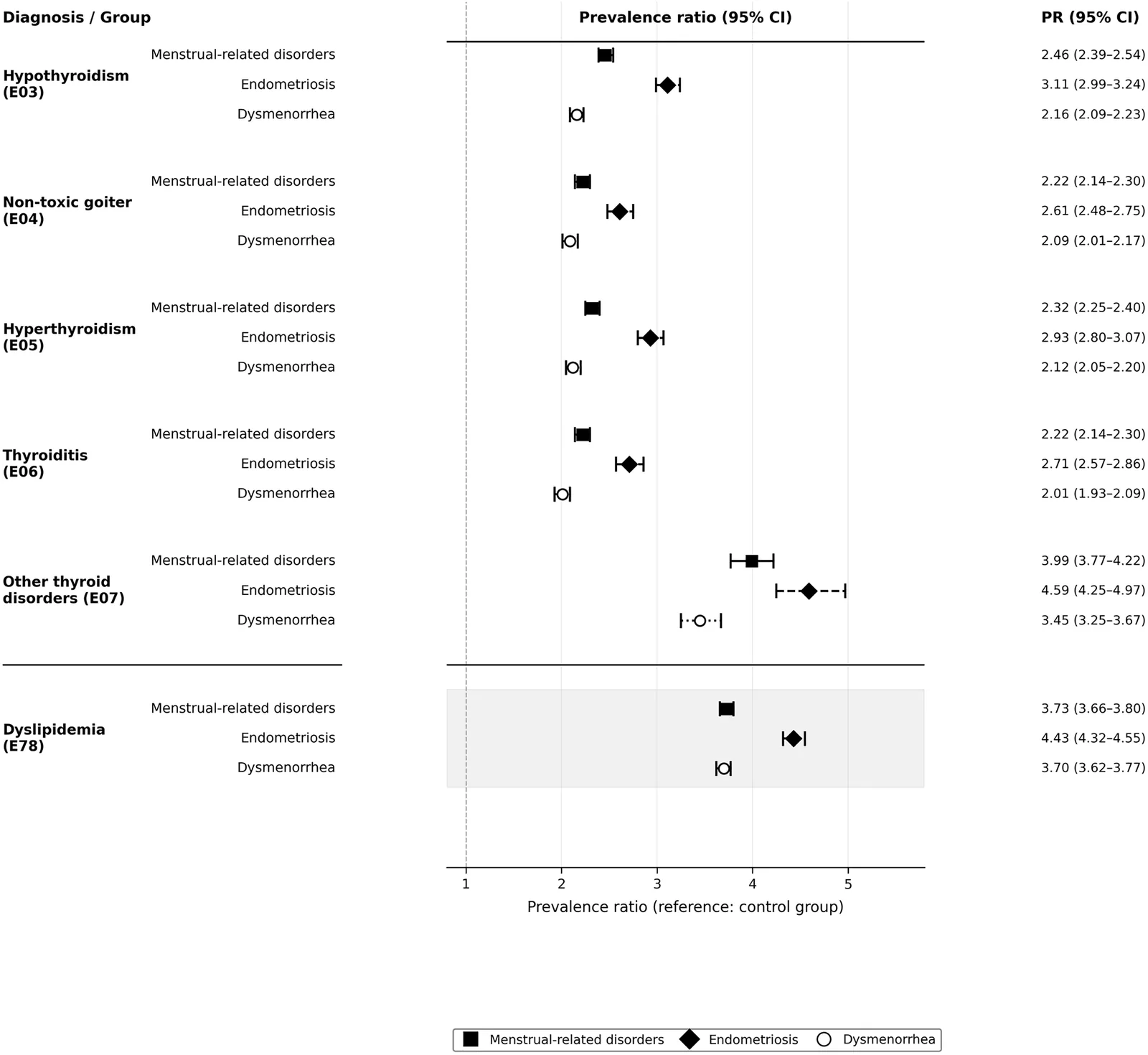
Prevalence ratios for thyroid-related diagnoses and dyslipidemia among women with menstrual-related disorders compared with controls. Prevalence ratios (PR) and 95% confidence intervals (CI) are shown for thyroid-related diagnoses and dyslipidemia in women with menstrual-related disorders, the endometriosis subgroup, and the dysmenorrhea subgroup, using women without menstrual-related disorders as the reference group. Thyroid-related diagnoses included hypothyroidism (E03), non-toxic goiter (E04), hyperthyroidism (E05), thyroiditis (E06), and other thyroid disorders (E07). Dyslipidemia was defined as ICD-10 code E78. Diagnosis categories may overlap within the same individual.

Hypothyroidism was identified in 5,322 women (2.1%) with menstrual-related disorders, 2,527 women (2.6%) in the endometriosis subgroup, and 4,300 women (1.8%) in the dysmenorrhea subgroup. In contrast, hypothyroidism was identified in 26,668 controls (0.8%). Using the control group as the reference, the PRs were 2.46 (95% CI, 2.39–2.54) for women with menstrual-related disorders, 3.11 (95% CI, 2.99–3.24) for the endometriosis subgroup, and 2.16 (95% CI, 2.09–2.23) for the dysmenorrhea subgroup.

Similarly, hyperthyroidism was identified in 4,108 women (1.6%) with menstrual-related disorders, 1,948 women (2.0%) in the endometriosis subgroup, and 3,466 women (1.5%) in the dysmenorrhea subgroup, compared with 21,846 controls (0.7%). The corresponding PRs were 2.32 (95% CI, 2.25–2.40), 2.93 (95% CI, 2.80–3.07), and 2.12 (95% CI, 2.05–2.20), respectively.

Non-toxic goiter, thyroiditis, and other thyroid disorders were also recorded more frequently among women with menstrual-related disorders and in each diagnostic subgroup than among controls. Overall, the frequencies of thyroid-related diagnoses tended to be highest in the endometriosis subgroup.

### Frequency of dyslipidemia

The frequency of dyslipidemia is shown in Table 2 and Fig 1. Comparisons for the endometriosis and dysmenorrhea subgroups are shown in S3 and S4 Tables. Dyslipidemia was identified in 13,331 women (5.2%) with menstrual-related disorders, 5,952 women (6.1%) in the endometriosis subgroup, and 12,176 women (5.1%) in the dysmenorrhea subgroup. In contrast, dyslipidemia was identified in 44,104 controls (1.4%).

**Table 2:** Frequency of dyslipidemia and association measures: Menstrual-related disorders vs. controls.

Diagnosis	Menstrual-related disordersn = 257,897	Controlsn = 3,183,032	PR(95% CI)	PD, percentage points(95% CI)
Dyslipidemia (E78)	13,331 (5.2)	44,104 (1.4)	3.73 (3.66 - 3.80)	+3.78 (+3.70 to +3.87)

Values are presented as n (%), prevalence ratio, and prevalence difference. The control group was used as the reference.

PR, prevalence ratio; PD, prevalence difference; CI, confidence interval.

Using the control group as the reference, the PRs for dyslipidemia were 3.73 (95% CI, 3.66–3.80) among women with menstrual-related disorders, 4.43 (95% CI, 4.32–4.55) in the endometriosis subgroup, and 3.70 (95% CI, 3.62–3.77) in the dysmenorrhea subgroup. The corresponding PDs were +3.78 percentage points (95% CI, + 3.70 to +3.87), + 4.76 percentage points (95% CI, + 4.61 to +4.91), and +3.74 percentage points (95% CI, + 3.65 to +3.83), respectively. The frequency of dyslipidemia was higher among women with menstrual-related disorders and in each diagnostic subgroup than among controls, with the highest frequency observed in the endometriosis subgroup.

### Age-adjusted analysis

To evaluate the influence of age confounding, we obtained age-stratified tabulations (six categories: < 15, 15–19, 20–24, 25–29, 30–34, and 35–39 years) for each disease outcome and exposure group, and calculated age-adjusted prevalence ratios using the Mantel-Haenszel method for risk ratios (Greenland–Robins estimator). The age distribution of each study group is shown in S5 Table, and the full age-stratified counts underlying the Mantel-Haenszel calculation are provided in S6 Table. The results of the age-adjusted analysis are shown in Table 3.

**Table 3:** Crude and age-adjusted prevalence ratios for thyroid-related diagnoses and dyslipidemia.

Diagnosis / Exposure group	Crude PR (95% CI)	MH-adjusted PR (95% CI)
Hypothyroidism (E03)		
Menstrual-related disorders	2.46 (2.39–2.54)	1.95 (1.89–2.00)
Endometriosis	3.11 (2.99–3.24)	2.18 (2.10–2.27)
Dysmenorrhea	2.16 (2.09–2.23)	1.76 (1.70–1.82)
Non-toxic goiter (E04)		
Menstrual-related disorders	2.22 (2.14–2.30)	1.55 (1.49–1.60)
Endometriosis	2.61 (2.48–2.75)	1.62 (1.54–1.70)
Dysmenorrhea	2.09 (2.01–2.17)	1.50 (1.44–1.55)
Hyperthyroidism (E05)		
Menstrual-related disorders	2.32 (2.25–2.40)	1.61 (1.55–1.66)
Endometriosis	2.93 (2.80–3.07)	1.83 (1.75–1.92)
Dysmenorrhea	2.12 (2.05–2.20)	1.51 (1.45–1.56)
Thyroiditis (E06)		
Menstrual-related disorders	2.22 (2.14–2.30)	1.55 (1.49–1.60)
Endometriosis	2.71 (2.57–2.86)	1.65 (1.56–1.74)
Dysmenorrhea	2.01 (1.93–2.09)	1.45 (1.39–1.51)
Other thyroid disorders (E07)		
Menstrual-related disorders	3.99 (3.77–4.22)	2.87 (2.71–3.03)
Endometriosis	4.59 (4.25–4.97)	3.02 (2.79–3.27)
Dysmenorrhea	3.45 (3.25–3.67)	2.53 (2.38–2.69)
Dyslipidemia (E78)		
Menstrual-related disorders	3.73 (3.66–3.80)	2.60 (2.55–2.65)
Endometriosis	4.43 (4.32–4.55)	2.74 (2.67–2.81)
Dysmenorrhea	3.70 (3.62–3.77)	2.65 (2.60–2.70)

Values are presented as crude prevalence ratios and Mantel-Haenszel age-adjusted prevalence ratios. The control group was used as the reference. Age adjustment was performed using six age strata (<15, 15–19, 20–24, 25–29, 30–34, and 35–39 years). Adjustment was limited to age; other potential confounders could not be adjusted for because individual-level data on these variables are not available in the JMDC Claims Database.

PR, prevalence ratio; MH, Mantel-Haenszel; CI, confidence interval.

After age adjustment, statistically significant associations were maintained across all 18 comparisons (six thyroid-related diagnoses and dyslipidemia × three exposure groups; menstrual-related disorders, endometriosis, and dysmenorrhea, each referenced against the control group), with adjusted prevalence ratios ranging from 1.45 to 3.02.”

### Descriptive comparison of healthcare utilization

For all six disease outcomes, women who received each diagnosis in the menstrual-related disorders group—including both the endometriosis and dysmenorrhea subgroups—had a higher number of annual outpatient visits than their counterparts in the control group (S7 Table). Across the six diagnoses, mean visit frequencies ranged from 18.2 to 23.1 in the menstrual-related disorders group and its subgroups, compared with 13.1 to 17.2 in the control group. This pattern was consistent across all outcomes and all subgroups, with the endometriosis subgroup generally showing the highest visit frequency. Among women who received each diagnosis, the mean number of annual outpatient visits was consistently higher in the LEP/DNG non-prescription group than in the prescription group across all six disease outcomes (differences ranging from 1.7 to 2.5 visits; S8 Table).

### Stratified analysis according to LEP/DNG prescription

The comparison according to LEP/DNG prescription status is shown in Table 4. Among women with menstrual-related disorders, 177,366 were included in the LEP/DNG prescription group and 80,531 were included in the non-prescription group.

**Table 4:** Frequency of thyroid-related diagnoses and dyslipidemia according to LEP/DNG prescription status among women with menstrual-related disorders.

Diagnosis	LEP/DNG prescription groupn = 177,366	LEP/DNG non-prescription groupn = 80,531	PR(95% CI)	PD, percentage points(95% CI)
Thyroid-related diagnoses				
Hypothyroidism (E03)	2,623 (1.5)	2,699 (3.4)	0.44 (0.42 - 0.47)	−1.87 (−2.01 to −1.74)
Non-toxic goiter (E04)	2,076 (1.2)	1,409 (1.7)	0.67 (0.63 - 0.72)	−0.58 (−0.68 to −0.48)
Hyperthyroidism (E05)	2,376 (1.3)	1,732 (2.2)	0.62 (0.59 - 0.66)	−0.81 (−0.92 to −0.70)
Thyroiditis (E06)	1,751 (1.0)	1,469 (1.8)	0.54 (0.51 - 0.58)	−0.84 (−0.94 to −0.73)
Other thyroid disorders (E07)	859 (0.5)	763 (0.9)	0.51 (0.46 - 0.56)	−0.46 (−0.54 to −0.39)
Dyslipidemia				
Dyslipidemia (E78)	9,231 (5.2)	4,100 (5.1)	1.02 (0.99 - 1.06)	+0.11 (−0.07 to +0.30)

Values are presented as n (%), prevalence ratios, and prevalence differences. The non-prescription group was used as the reference. Thyroid-related diagnosis codes may overlap within the same individual.

LEP, low-dose estrogen–progestin preparations; DNG, dienogest; PR, prevalence ratio; PD, prevalence difference; CI, confidence interval.

Hypothyroidism was identified in 2,623 women (1.5%) in the LEP/DNG prescription group and 2,699 women (3.4%) in the non-prescription group. Thyroiditis was also more frequent in the non-prescription group than in the prescription group (1.8% vs. 1.0%).

In contrast, dyslipidemia was identified in 9,231 women (5.2%) in the LEP/DNG prescription group and 4,100 women (5.1%) in the non-prescription group. There was no substantial difference in the frequency of dyslipidemia between the two groups (PR, 1.02; 95% CI, 0.99–1.06).

## Discussion

In this nationwide claims-based study of Japanese women aged <40 years, we investigated the association of menstrual-related disorders with the frequencies of thyroid-related diagnoses and dyslipidemia. The main finding was that women with menstrual-related disorders had higher frequencies of thyroid-related diagnoses and dyslipidemia than controls. These findings should be interpreted as co-recorded diagnoses in claims data rather than as evidence of incident disease risk. This tendency was most pronounced in the endometriosis subgroup but was also consistently observed in the dysmenorrhea subgroup. Notably, these associations remained statistically significant after adjustment for age using the Mantel-Haenszel method.

The present findings are consistent with the emerging concept that endometriosis is not merely a pelvic disease but a systemic disorder involving immune, inflammatory, and endocrine-metabolic alterations [9,23,24]. The association between endometriosis and autoimmune diseases, including autoimmune thyroid disease, has been examined in systematic reviews and meta-analyses [11,12,20]. In addition, recent large-scale retrospective cohort studies have reported increased risks of thyroid disorders, including hypothyroidism, hyperthyroidism, Graves’ disease, and Hashimoto’s thyroiditis, after a diagnosis of endometriosis. [21,22,25]. In the present study, hypothyroidism, hyperthyroidism, thyroiditis, and non-toxic goiter were all more frequently recorded among women with menstrual-related disorders than among controls. The particularly high frequencies observed in the endometriosis subgroup are consistent with these previous findings.

Although endometriosis should not be simply classified as an autoimmune disease, prior literature has noted that endometriosis frequently co-occurs with autoinflammatory and autoimmune conditions [12,26–28]. The higher frequency of thyroid-related diagnoses observed among women with menstrual-related disorders, particularly in the endometriosis subgroup, is consistent with this reported co-occurrence, although the present cross-sectional design does not allow us to determine whether this reflects a shared underlying process or independent co-recorded diagnoses.

Dyslipidemia was also more frequently recorded among women with menstrual-related disorders than among controls. Prior genetic epidemiological studies examining serum lipids or the lipidome in relation to female reproductive disorders, including endometriosis, have reported associations between altered lipid profiles and endometriosis [29–32]. Dyslipidemia is also associated with long-term cardiovascular risk, making the recognition of this co-recorded diagnosis in young women clinically relevant [18,33]. However, because this was a cross-sectional study using claims data, the present findings represent co-recorded diagnoses rather than evidence of a causal or temporal relationship. We could not determine whether dyslipidemia preceded menstrual-related disorders, was diagnosed during the course of medical care for menstrual-related disorders, or was explained by shared background factors.

A novel aspect of this study is that higher frequencies of thyroid-related diagnoses and dyslipidemia were observed not only in the endometriosis subgroup but also in the dysmenorrhea subgroup. Most previous studies have focused on endometriosis, but recent reports suggest that dysmenorrhea and menstrual irregularities may also be associated with future cardiovascular disease risk [15–35]. Dysmenorrhea includes heterogeneous conditions such as primary dysmenorrhea, undiagnosed endometriosis, adenomyosis, pelvic inflammation, and functional pain [3,36]. Therefore, the associations observed in this study may not be limited to histologically confirmed endometriosis but may reflect endocrine and metabolic comorbidities in a broader population of young women with menstrual pain.

In the stratified analysis according to LEP/DNG prescription status, thyroid-related diagnoses were more frequent in the non-prescription group than in the prescription group. However, these findings should not be interpreted as evidence of a pharmacological effect of LEP/DNG on thyroid function. Because LEP/DNG prescription reflects routine clinical decision-making rather than random assignment, differences between the prescription and non-prescription groups may have been influenced by patient characteristics that were not fully captured in the present study. One possible explanation is that treatment decisions were shaped by the overall clinical profile of individual patients, including symptom severity, comorbidities, contraindications, physician judgment, patient preference, and reproductive plans, resulting in systematic differences between women who did and did not receive LEP/DNG. Consistent with this possibility, among women diagnosed with each endocrine disorder, the mean number of annual outpatient visits was consistently higher in the non-prescription group than in the prescription group across all six disease outcomes examined. This pattern may suggest that women requiring more frequent medical care, potentially reflecting greater disease burden or more complex clinical management, were somewhat less likely to receive LEP/DNG. Taken together, these observations raise the possibility that the observed differences primarily reflect confounding by indication rather than a direct treatment effect. Therefore, the comparison according to LEP/DNG prescription status should be interpreted as descriptive of real-world prescribing patterns rather than as an evaluation of treatment effects.

On the other hand, hormonal therapy, particularly estrogen–progestin preparations, may affect lipid metabolism [37,38]. Previous studies have reported that oral contraceptive use can alter lipid profiles, including total cholesterol, low-density lipoprotein cholesterol (LDL-C), triglycerides, and high-density lipoprotein cholesterol (HDL-C) [37,38]. However, these effects may vary according to estrogen dose, progestin type, route of administration, duration of use, and baseline cardiometabolic risk [39]. In the present study, there was no substantial difference in the frequency of dyslipidemia between women with and without LEP/DNG prescriptions. Nevertheless, because claims data do not include measured lipid values or longitudinal changes before and after treatment initiation, we could not assess the direct effects of LEP or DNG on lipid metabolism. Therefore, this analysis should not be interpreted as an evaluation of the metabolic safety of these medications.

The strengths of this study include its large-scale analysis using nationwide claims data comprising more than 3.4 million women. By focusing on women aged <40 years and evaluating not only endometriosis but also dysmenorrhea, this study broadly captured menstrual-related disorders commonly encountered in real-world clinical practice. Another strength is the simultaneous evaluation of thyroid-related diagnoses and dyslipidemia within the same database.

This study has several important limitations. First, because of its cross-sectional design, temporal and causal relationships between menstrual-related disorders and thyroid-related diagnoses or dyslipidemia could not be assessed. Second, because diagnoses were based on claims data, laboratory values such as thyroid-stimulating hormone, free thyroxine, thyroid autoantibodies, LDL-C, HDL-C, and triglycerides, as well as imaging findings and symptom severity, were unavailable. Third, surveillance bias cannot be excluded. Women with menstrual-related disorders generally have more frequent contact with the healthcare system, which may increase the opportunity for incidental diagnosis of thyroid disorders and dyslipidemia. In a descriptive comparison restricted to women who received each diagnosis, the mean number of annual outpatient visits was consistently higher in the menstrual-related disorders group and its subgroups than in the control group across all six disease outcomes examined. This consistent pattern suggests that surveillance bias may have contributed, at least in part, to the associations observed across all outcomes in this study. Because individual-level data linking outpatient visit frequency to disease-free status were not available, a formal adjustment for healthcare utilization could not be performed. The crude and age-adjusted prevalence ratios reported here should therefore be interpreted as reflecting a combination of a true association and the influence of differential surveillance. Fourth, this study was based on aggregated tabular data rather than individual-level records. Consequently, important confounders such as body mass index, smoking, alcohol consumption, family history, pregnancy history, and socioeconomic status could not be adjusted for, and a multivariable model simultaneously adjusting for age and healthcare utilization could not be constructed. This is a structural limitation of the aggregated data format used in this study rather than a limitation that could be resolved through additional tabulation requests. The age-adjusted analysis and the descriptive comparison of healthcare utilization presented in this study should therefore be regarded as complementary but independent analyses. Studies with access to individual-level data would be needed to perform multivariable adjustment for these factors simultaneously. Fifth, diagnoses of endometriosis and dysmenorrhea were based on ICD-10 codes and could not be confirmed by laparoscopy, magnetic resonance imaging, or ultrasonography. The validity of administrative data for identifying endometriosis has been examined in prior validation studies: a study linking surgically confirmed endometriosis cases to administrative records reported a sensitivity of 0.88, specificity of 0.87, and substantial agreement (κ = 0.74), although sensitivity was considerably lower for deep infiltrating endometriosis (0.12) [40]. A separate validation study using the Danish National Patient Registry reported a positive predictive value of 94.9% (95% CI, 91.7–97.1) for endometriosis diagnosis codes [41]. Validation evidence for dysmenorrhea coding is more limited, as this diagnosis is largely symptom-based rather than confirmed by imaging or surgery, and misclassification cannot be excluded. Sixth, dyslipidemia (E78) reflects physician-diagnosed and recorded dyslipidemia rather than measured lipid abnormalities such as LDL-C, HDL-C, or triglycerides. Asymptomatic young women with elevated LDL-C often do not receive a formal ICD diagnosis in the absence of clinical intervention, which would be expected to bias the observed frequency of E78 toward underestimation in both the menstrual-related disorders and control groups. Finally, thyroid-related diagnosis codes may overlap within the same individual. Therefore, the simple sum of E03–E07 codes does not represent the true individual-level prevalence of any thyroid disease after removal of duplicate diagnoses.

In conclusion, this nationwide claims-based study showed that Japanese women aged <40 years with menstrual-related disorders had higher frequencies of thyroid-related diagnoses and dyslipidemia than controls. This tendency was particularly pronounced in the endometriosis subgroup. Although this study does not establish causality, the findings suggest the potential importance of considering possible endocrine and metabolic comorbidities in young women with menstrual-related disorders.

## Supporting information

S1 Table Frequency of thyroid-related diagnoses: Endometriosis vs. controls. (DOCX)

S2 Table Frequency of thyroid-related diagnoses: Dysmenorrhea vs. controls. (DOCX)

S3 Table Frequency of dyslipidemia and association measures: Endometriosis subgroup vs. controls. (DOCX)

S4 Table Frequency of dyslipidemia and association measures: Dysmenorrhea subgroup vs. controls. (DOCX)

S5 Table Age distribution of study groups. (DOCX)

S6 Table Age-stratified case counts underlying the Mantel-Haenszel analysis. (DOCX)

S7 Table Outpatient visit frequency among women diagnosed with each disease outcome, by exposure group. (DOCX)

S8 Table Outpatient visit frequency among women diagnosed with each disease outcome, by LEP/DNG prescription status among women with menstrual-related disorders. (DOCX)

S1 Checklist STROBE checklist for cross-sectional studies. (DOCX)
