# Supplementary material for: Frequencies of thyroid-related diagnoses and dyslipidemia among Japanese women with menstrual-related disorders aged under 40 years: A nationwide cross-sectional study using health insurance claims data
Source: PLoS One. 2026 Oct 30;21(10):e0361575. doi: 10.1371/journal.pone.0361575 (PMC13722161; doi:10.1371/journal.pone.0361575)
Supplement: Supplementary file 1 [file pone.0361575.s001.docx]

S1 Table. Frequency of thyroid-related diagnoses: endometriosis vs. controls

| Thyroid-related diagnosis | Endometriosis n = 96,863 | Controls n = 3,183,032 | PR (95% CI) | PD, percentage points (95% CI) |
| --- | --- | --- | --- | --- |
| Hypothyroidism (E03) | 2,527 (2.6) | 26,668 (0.8) | 3.11 (2.99 - 3.24) | +1.77 (+1.67 to +1.87) |
| Non-toxic goiter (E04) | 1,536 (1.6) | 19,360 (0.6) | 2.61 (2.48 - 2.75) | +0.98 (+0.90 to +1.06) |
| Hyperthyroidism (E05) | 1,948 (2.0) | 21,846 (0.7) | 2.93 (2.80 - 3.07) | +1.32 (+1.24 to +1.41) |
| Thyroiditis (E06) | 1,480 (1.5) | 17,922 (0.6) | 2.71 (2.57 - 2.86) | +0.96 (+0.89 to +1.04) |
| Other thyroid disorders (E07) | 702 (0.7) | 5,021 (0.2) | 4.59 (4.25 - 4.97) | +0.57 (+0.51 to +0.62) |

Values are presented as n (%), prevalence ratios, and prevalence differences. The control group was used as the reference. Thyroid-related diagnosis codes may overlap within the same individual; therefore, the sum of E03–E07 codes does not represent the individual-level prevalence of any thyroid disease after duplicate diagnoses are removed.

PR, prevalence ratio; PD, prevalence difference; CI, confidence interval
