# Supplementary material for: Frequencies of thyroid-related diagnoses and dyslipidemia among Japanese women with menstrual-related disorders aged under 40 years: A nationwide cross-sectional study using health insurance claims data
Source: PLoS One. 2026 Oct 30;21(10):e0361575. doi: 10.1371/journal.pone.0361575 (PMC13722161; doi:10.1371/journal.pone.0361575)
Supplement: Supplementary file 2 [file pone.0361575.s002.docx]

S2 Table. Frequency of thyroid-related diagnoses: dysmenorrhea vs. controls

| Thyroid-related diagnosis | Dysmenorrhea n = 237,727 | Controls n = 3,183,032 | PR (95% CI) | PD, percentage points (95% CI) |
| --- | --- | --- | --- | --- |
| Hypothyroidism (E03) | 4,300 (1.8) | 26,668 (0.8) | 2.16 (2.09 - 2.23) | +0.97 (+0.92 to +1.03) |
| Non-toxic goiter (E04) | 3,017 (1.3) | 19,360 (0.6) | 2.09 (2.01 - 2.17) | +0.66 (+0.62 to +0.71) |
| Hyperthyroidism (E05) | 3,466 (1.5) | 21,846 (0.7) | 2.12 (2.05 - 2.20) | +0.77 (+0.72 to +0.82) |
| Thyroiditis (E06) | 2,691 (1.1) | 17,922 (0.6) | 2.01 (1.93 - 2.09) | +0.57 (+0.53 to +0.61) |
| Other thyroid disorders (E07) | 1,294 (0.5) | 5,021 (0.2) | 3.45 (3.25 - 3.67) | +0.39 (+0.36 to +0.42) |

Values are presented as n (%), prevalence ratios, and prevalence differences. The control group was used as the reference. Thyroid-related diagnosis codes may overlap within the same individual; therefore, the sum of E03–E07 codes does not represent the individual-level prevalence of any thyroid disease after duplicate diagnoses are removed.

PR, prevalence ratio; PD, prevalence difference; CI, confidence interval
