# Supplementary material for: Frequencies of thyroid-related diagnoses and dyslipidemia among Japanese women with menstrual-related disorders aged under 40 years: A nationwide cross-sectional study using health insurance claims data
Source: PLoS One. 2026 Oct 30;21(10):e0361575. doi: 10.1371/journal.pone.0361575 (PMC13722161; doi:10.1371/journal.pone.0361575)
Supplement: Supplementary file 3 [file pone.0361575.s003.docx]

S3 Table. Frequency of dyslipidemia and association measures: endometriosis subgroup vs. controls

| Diagnosis | Endometriosis n = 96,863 | Controls n = 3,183,032 | PR (95% CI) | PD, percentage points (95% CI) |
| --- | --- | --- | --- | --- |
| Dyslipidemia (E78) | 5,952 (6.1) | 44,104 (1.4) | 4.43 (4.32 - 4.55) | +4.76 (+4.61 to +4.91) |

Values are presented as n (%), prevalence ratio, and prevalence difference. The control group was used as the reference.

PR, prevalence ratio; PD, prevalence difference; CI, confidence interval
