# Supplementary material for: Frequencies of thyroid-related diagnoses and dyslipidemia among Japanese women with menstrual-related disorders aged under 40 years: A nationwide cross-sectional study using health insurance claims data
Source: PLoS One. 2026 Oct 30;21(10):e0361575. doi: 10.1371/journal.pone.0361575 (PMC13722161; doi:10.1371/journal.pone.0361575)
Supplement: Supplementary file 4 [file pone.0361575.s004.docx]

S4 Table. Frequency of dyslipidemia and association measures: dysmenorrhea subgroup vs. controls

| Diagnosis | Dysmenorrhea n = 237,727 | Controls n = 3,183,032 | PR (95% CI) | PD, percentage points (95% CI) |
| --- | --- | --- | --- | --- |
| Dyslipidemia (E78) | 12,176 (5.1) | 44,104 (1.4) | 3.70 (3.62 - 3.77) | +3.74 (+3.65 to +3.83) |

Values are presented as n (%), prevalence ratio, and prevalence difference. The control group was used as the reference.

PR, prevalence ratio; PD, prevalence difference; CI, confidence interval
