# Supplementary material for: Frequencies of thyroid-related diagnoses and dyslipidemia among Japanese women with menstrual-related disorders aged under 40 years: A nationwide cross-sectional study using health insurance claims data
Source: PLoS One. 2026 Oct 30;21(10):e0361575. doi: 10.1371/journal.pone.0361575 (PMC13722161; doi:10.1371/journal.pone.0361575)
Supplement: Supplementary file 5 [file pone.0361575.s005.docx]

S5 Table. Age distribution of study groups

| Age group | Menstrual-related disorders n (%) | Endometriosis n (%) | Dysmenorrhea n (%) | Controls n (%) |
| --- | --- | --- | --- | --- |
| <15 years | 8,496 (3.3) | 1,040 (1.1) | 8,378 (3.5) | 1,138,509 (35.8) |
| 15–19 years | 29,723 (11.5) | 6,835 (7.1) | 29,253 (12.3) | 377,987 (11.9) |
| 20–24 years | 62,775 (24.3) | 19,737 (20.4) | 60,951 (25.6) | 434,965 (13.7) |
| 25–29 years | 60,858 (23.6) | 23,259 (24.0) | 57,145 (24.0) | 396,294 (12.5) |
| 30–34 years | 47,958 (18.6) | 21,256 (21.9) | 42,014 (17.7) | 395,569 (12.4) |
| 35–39 years | 48,087 (18.6) | 24,736 (25.5) | 39,986 (16.8) | 439,708 (13.8) |
| Total | 257,897 (100) | 96,863 (100) | 237,727 (100) | 3,183,032 (100) |

Age was available only as a categorical variable; individual-level continuous age data were not available.
