# Supplementary material for: Frequencies of thyroid-related diagnoses and dyslipidemia among Japanese women with menstrual-related disorders aged under 40 years: A nationwide cross-sectional study using health insurance claims data
Source: PLoS One. 2026 Oct 30;21(10):e0361575. doi: 10.1371/journal.pone.0361575 (PMC13722161; doi:10.1371/journal.pone.0361575)
Supplement: Supplementary file 6 [file pone.0361575.s006.docx]

S6 Table. Age-stratified case counts underlying the Mantel-Haenszel analysis

E03 Hypothyroidism

| Age group | Menstrual-related disorders | Endometriosis | Dysmenorrhea | Controls |
| --- | --- | --- | --- | --- |
| <15 years | 121 | 21 | 117 | 4,272 |
| 15–19 years | 419 | 100 | 415 | 1,946 |
| 20–24 years | 723 | 264 | 692 | 2,245 |
| 25–29 years | 995 | 448 | 856 | 3,719 |
| 30–34 years | 1,387 | 730 | 996 | 6,532 |
| 35–39 years | 1,677 | 964 | 1,224 | 7,954 |

Non-toxic goiter (E04)

| Age group | Menstrual-related disorders | Endometriosis | Dysmenorrhea | Controls |
| --- | --- | --- | --- | --- |
| <15 years | 44 | 5 | 44 | 906 |
| 15–19 years | 215 | 47 | 214 | 1,603 |
| 20–24 years | 500 | 156 | 485 | 2,253 |
| 25–29 years | 718 | 291 | 656 | 3,252 |
| 30–34 years | 880 | 422 | 727 | 4,808 |
| 35–39 years | 1,128 | 615 | 891 | 6,538 |

Hyperthyroidism (E05)

| Age group | Menstrual-related disorders | Endometriosis | Dysmenorrhea | Controls |
| --- | --- | --- | --- | --- |
| <15 years | 64 | 19 | 60 | 1,078 |
| 15–19 years | 360 | 119 | 347 | 1,799 |
| 20–24 years | 679 | 251 | 646 | 2,761 |
| 25–29 years | 865 | 396 | 749 | 4,060 |
| 30–34 years | 1,007 | 512 | 804 | 5,593 |
| 35–39 years | 1,133 | 651 | 860 | 6,555 |

Thyroiditis (E06)

| Age group | Menstrual-related disorders | Endometriosis | Dysmenorrhea | Controls |
| --- | --- | --- | --- | --- |
| <15 years | 29 | 6 | 28 | 766 |
| 15–19 years | 172 | 37 | 170 | 1,180 |
| 20–24 years | 371 | 109 | 355 | 1,652 |
| 25–29 years | 609 | 265 | 540 | 2,847 |
| 30–34 years | 874 | 431 | 703 | 4,829 |
| 35–39 years | 1,165 | 632 | 895 | 6,648 |

Other thyroid disorders (E07)

| Age group | Menstrual-related disorders | Endometriosis | Dysmenorrhea | Controls |
| --- | --- | --- | --- | --- |
| <15 years | 35 | 5 | 34 | 456 |
| 15–19 years | 114 | 23 | 110 | 450 |
| 20–24 years | 296 | 86 | 282 | 602 |
| 25–29 years | 383 | 174 | 309 | 965 |
| 30–34 years | 389 | 196 | 276 | 1,244 |
| 35–39 years | 405 | 218 | 283 | 1,304 |

Dyslipidemia (E78)

| Age group | Menstrual-related disorders | Endometriosis | Dysmenorrhea | Controls |
| --- | --- | --- | --- | --- |
| <15 years | 135 | 27 | 133 | 2,434 |
| 15–19 years | 911 | 324 | 894 | 2,633 |
| 20–24 years | 2,536 | 968 | 2,469 | 5,436 |
| 25–29 years | 2,995 | 1,288 | 2,835 | 7,405 |
| 30–34 years | 3,025 | 1,394 | 2,687 | 10,227 |
| 35–39 years | 3,729 | 1,951 | 3,158 | 15,969 |

Denominators (total number of women in each age stratum and group) are provided in S5 Table. These counts were used to calculate the Mantel-Haenszel age-adjusted prevalence ratios presented in Table 3.
