# Supplementary material for: Frequencies of thyroid-related diagnoses and dyslipidemia among Japanese women with menstrual-related disorders aged under 40 years: A nationwide cross-sectional study using health insurance claims data
Source: PLoS One. 2026 Oct 30;21(10):e0361575. doi: 10.1371/journal.pone.0361575 (PMC13722161; doi:10.1371/journal.pone.0361575)
Supplement: Supplementary file 7 [file pone.0361575.s007.docx]

S7 Table. Outpatient visit frequency among women diagnosed with each disease outcome, by exposure group

| Diagnosis | Group | Mean | SD | Median | IQR |
| --- | --- | --- | --- | --- | --- |
| Hypothyroidism (E03) | Menstrual-related disorders | 21.9 | 16.3 | 18 | 10–29 |
|  | Endometriosis | 23.1 | 16.8 | 19 | 11–31 |
|  | Dysmenorrhea | 21.2 | 16.4 | 17 | 10–28 |
|  | Controls | 17.2 | 14.8 | 13 | 7–22 |
| Non-toxic goiter (E04) | Menstrual-related disorders | 19.9 | 14.3 | 16 | 10–26 |
|  | Endometriosis | 20.6 | 14.1 | 17 | 11–26 |
|  | Dysmenorrhea | 19.5 | 14.1 | 16 | 10–25 |
|  | Controls | 13.1 | 12.0 | 10 | 5–17 |
| Hyperthyroidism (E05) | Menstrual-related disorders | 19.5 | 13.5 | 16 | 10–25 |
|  | Endometriosis | 20.1 | 13.9 | 17 | 10–26 |
|  | Dysmenorrhea | 19.2 | 13.3 | 16 | 10–25 |
|  | Controls | 13.6 | 11.3 | 11 | 6–18 |
| Thyroiditis (E06) | Menstrual-related disorders | 21.0 | 14.9 | 17 | 11–27 |
|  | Endometriosis | 22.6 | 15.9 | 18 | 11–29 |
|  | Dysmenorrhea | 20.4 | 14.6 | 16 | 10–26 |
|  | Controls | 14.3 | 12.7 | 11 | 6–18 |
| Other thyroid disorders (E07) | Menstrual-related disorders | 20.6 | 15.7 | 17 | 10–27 |
|  | Endometriosis | 22.2 | 15.6 | 19 | 11–29 |
|  | Dysmenorrhea | 19.8 | 15.7 | 16 | 9–26 |
|  | Controls | 17.1 | 14.7 | 13 | 7–23 |
| Dyslipidemia (E78) | Menstrual-related disorders | 18.2 | 14.9 | 14 | 9–23 |
|  | Endometriosis | 18.8 | 15.9 | 15 | 9–24 |
|  | Dysmenorrhea | 18.0 | 14.8 | 14 | 9–23 |
|  | Controls | 13.4 | 13.0 | 10 | 5–17 |

Values represent the annual number of outpatient visits among women who received each diagnosis, stratified by exposure group. Data were not available for women without each diagnosis; therefore, a formal stratified adjustment for healthcare utilization could not be performed.

SD, standard deviation; IQR, interquartile range.
