# Supplementary material for: Frequencies of thyroid-related diagnoses and dyslipidemia among Japanese women with menstrual-related disorders aged under 40 years: A nationwide cross-sectional study using health insurance claims data
Source: PLoS One. 2026 Oct 30;21(10):e0361575. doi: 10.1371/journal.pone.0361575 (PMC13722161; doi:10.1371/journal.pone.0361575)
Supplement: Supplementary file 8 [file pone.0361575.s008.docx]

S8 Table. Outpatient visit frequency among women diagnosed with each disease outcome, by LEP/DNG prescription status among women with menstrual-related disorders

| Diagnosis | Group | Mean | SD | Median | IQR |
| --- | --- | --- | --- | --- | --- |
| Hypothyroidism (E03) | LEP/DNG prescription | 20.8 | 16.1 | 17 | 10–27 |
|  | LEP/DNG non-prescription | 23.1 | 16.5 | 19 | 11–31 |
| Non-toxic goiter (E04) | LEP/DNG prescription | 19.0 | 13.1 | 15 | 10–24 |
|  | LEP/DNG non-prescription | 21.3 | 15.8 | 17 | 10–28 |
| Hyperthyroidism (E05) | LEP/DNG prescription | 18.8 | 12.7 | 16 | 10–24 |
|  | LEP/DNG non-prescription | 20.5 | 14.5 | 17 | 10–27 |
| Thyroiditis (E06) | LEP/DNG prescription | 20.1 | 14.3 | 16 | 11–25 |
|  | LEP/DNG non-prescription | 22.1 | 15.5 | 18 | 11–30 |
| Other thyroid disorders (E07) | LEP/DNG prescription | 19.4 | 15.8 | 15 | 9–25 |
|  | LEP/DNG non-prescription | 21.9 | 15.5 | 19 | 10–30 |
| Dyslipidemia (E78) | LEP/DNG prescription | 17.6 | 14.4 | 14 | 9–22 |
|  | LEP/DNG non-prescription | 19.6 | 15.9 | 15 | 9–26 |

Values represent the annual number of outpatient visits among women who received each diagnosis, stratified by LEP/DNG prescription status, among women with menstrual-related disorders. Data were not available for women without each diagnosis; therefore, a formal stratified adjustment for healthcare utilization could not be performed.
