# Supplementary material for: Frequencies of thyroid-related diagnoses and dyslipidemia among Japanese women with menstrual-related disorders aged under 40 years: A nationwide cross-sectional study using health insurance claims data
Source: PLoS One. 2026 Oct 30;21(10):e0361575. doi: 10.1371/journal.pone.0361575 (PMC13722161; doi:10.1371/journal.pone.0361575)
Supplement: Supplementary file 9 [file pone.0361575.s009.docx]

**STROBE Statement — Checklist of items that should be included in reports of cross-sectional studies**

**Manuscript title:** Association of menstrual-related disorders with thyroid-related diagnoses and dyslipidemia among Japanese women aged under 40 years: a nationwide cross-sectional study using health insurance claims data

**Journal:** PLOS ONE

| **Section** | **Item** | **Recommendation** | **Reported on page / line No. (or comment)** |
| --- | --- | --- | --- |
| **TITLE AND ABSTRACT** | | | |
| Title and abstract | **1a** | (a) Indicate the study's design with a commonly used term in the title or the abstract | Title (page 1): "...a nationwide cross-sectional study..." |
|  | **1b** | (b) Provide in the abstract an informative and balanced summary of what was done and what was found | Abstract (page 2): study design, population, outcomes, main results, and limitations stated |
| **INTRODUCTION** | | | |
| Background / rationale | **2** | Explain the scientific background and rationale for the investigation being reported | Introduction, paragraphs 1–3 (page 3) |
| Objectives | **3** | State specific objectives, including any prespecified hypotheses | Introduction, final paragraph (page 3): objectives of the study stated explicitly |
| **METHODS** | | | |
| Study design | **4** | Present key elements of study design early in the paper | Methods – Study design and data source (page 4): "This was a nationwide cross-sectional study using health insurance claims data." |
| Setting | **5** | Describe the setting, locations, and relevant dates, including periods of recruitment, exposure, follow-up, and data collection | Methods – Study design and data source (page 4): JMDC Claims Database; observation period January–December 2023; Japan |
| Participants | **6** | (a) Give the eligibility criteria, and the sources and methods of selection of participants | Methods – Study population (page 4): women aged <40 years enrolled in JMDC in 2023; confirmed diagnoses of N80, N94.4, or N94.6 for the menstrual-related disorders group; absence of such diagnoses for controls |
| Variables | **7** | Clearly define all outcomes, exposures, predictors, potential confounders, and effect modifiers. Give diagnostic criteria, if applicable | Methods – Outcomes (page 4–5): primary outcomes defined by ICD-10 codes (E03–E07, E78). Exposure (LEP/DNG prescription) defined in Methods – Hormonal therapy (page 5). Confounders not adjusted for (acknowledged as limitation) |
| Data sources / measurement | **8*** | For each variable of interest, give sources of data and details of methods of assessment (measurement). Describe comparability of assessment methods if there is more than one group | Methods – Study design and data source (page 4): diagnoses coded by ICD-10; prescriptions identified by drug codes from the JMDC database. Same coding system applied to all groups |
| Bias | **9** | Describe any efforts to address potential sources of bias | Discussion – Limitations (page 10): surveillance bias (women with MRD have more frequent visits), confounding by indication (LEP/DNG), and age confounding discussed. No active bias-correction was applied; limitations acknowledged |
| Study size | **10** | Explain how the study size was arrived at | N/A – All eligible women in the JMDC database during 2023 were included (total population analysis). No a priori sample size calculation was performed |
| Quantitative variables | **11** | Explain how quantitative variables were handled in the analyses. If applicable, describe which groupings were chosen and why | Methods – Study population (page 4): age categorised into six groups (<15, 15–19, 20–24, 25–29, 30–34, 35–39 years). All primary outcomes are binary (diagnosed / not diagnosed) |
| Statistical methods | **12a** | (a) Describe all statistical methods, including those used to control for confounding | Methods – Statistical analysis (page 5): prevalence ratios (PR) and prevalence differences (PD) with 95% CIs calculated. PR 95% CI by log-transformation delta method; PD 95% CI by standard error for difference between two proportions. Python 3.12.0 used. No multivariable adjustment (acknowledged as limitation) |
|  | **12b** | (b) Describe any methods used to examine subgroups and interactions | Methods – Statistical analysis (page 5): pre-specified subgroup analyses for endometriosis and dysmenorrhea subgroups; stratified analysis by LEP/DNG prescription status |
|  | **12c** | (c) Explain how missing data were addressed | N/A – Claims database; diagnoses not recorded are treated as absent. Missing data not applicable in this setting (acknowledged implicitly) |
|  | **12d** | (d) If applicable, describe analytical methods taking account of sampling strategy | N/A – Total population analysis; no sampling was performed |
|  | **12e** | (e) Describe any sensitivity analyses | N/A – Formal sensitivity analyses were not conducted. Limitations section (page 10) addresses potential biases qualitatively. Future sensitivity analysis restricting to age 15–39 years noted as needed |
| **RESULTS** | | | |
| Participants | **13a*** | (a) Report numbers of individuals at each stage of study | Results – Study population (page 6): total N = 3,440,929; MRD group n = 257,897; endometriosis subgroup n = 96,863; dysmenorrhea subgroup n = 237,727; control group n = 3,183,032; LEP/DNG prescription group n = 177,366 |
|  | **13b*** | (b) Give reasons for non-participation at each stage | N/A – All eligible women in the database were included; no refusals or dropouts |
|  | **13c*** | (c) Consider use of a flow diagram | N/A – Not included. Study population described in text (page 6) and Table 1 |
| Descriptive data | **14a*** | (a) Give characteristics of study participants (demographic, clinical, social) and information on exposures and potential confounders | Results – Study population (page 6) and Table 1: age distribution and LEP/DNG prescription status reported for all groups |
|  | **14b*** | (b) Indicate number of participants with missing data for each variable of interest | N/A – Claims data; no missing data for the variables used (ICD-10 diagnoses and drug codes) |
| Outcome data | **15*** | Report numbers of outcome events or summary measures | Results (pages 6–8), Tables 1–3, Supplementary Tables 1–4: n and % for each thyroid-related diagnosis and dyslipidemia reported for all groups |
| Main results | **16a** | (a) Give unadjusted estimates and, if applicable, confounder-adjusted estimates and their precision (95% CI) | Results (pages 6–8) and Tables 1–3: crude PR with 95% CI and PD with 95% CI reported. Confounder-adjusted estimates not calculated (limitation acknowledged) |
|  | **16b** | (b) Report category boundaries when continuous variables were categorized | Methods – Study population (page 4): age categories defined (<15, 15–19, 20–24, 25–29, 30–34, 35–39 years) |
|  | **16c** | (c) If relevant, consider translating estimates of relative risk into absolute risk for a meaningful time period | Results (pages 7–8): prevalence differences (absolute risk differences) reported alongside PRs for all primary outcomes |
| Other analyses | **17** | Report other analyses done—e.g., analyses of subgroups and interactions, and sensitivity analyses | Results (pages 7–8) and Table 3, Supplementary Tables 1–4: pre-specified subgroup analyses (endometriosis, dysmenorrhea) and stratified analysis by LEP/DNG prescription status reported |
| **DISCUSSION** | | | |
| Key results | **18** | Summarise key results with reference to study objectives | Discussion, first paragraph (page 8): key findings summarised with reference to study objectives |
| Limitations | **19** | Discuss limitations of the study, taking into account sources of potential bias or imprecision. Discuss both direction and magnitude of any potential bias | Discussion – Limitations (pages 9–10): cross-sectional design, claims-based diagnoses, unmeasured confounders (BMI, smoking, family history), surveillance bias, confounding by indication (LEP/DNG), age confounding (control group age composition), ICD-10 code overlap for thyroid diagnoses |
| Interpretation | **20** | Give a cautious overall interpretation of results considering objectives, limitations, multiplicity of analyses, results from similar studies, and other relevant evidence | Discussion (pages 8–10): results interpreted as co-recorded diagnoses, not incident disease risk; findings contextualised against prior literature; causality explicitly not claimed |
| Generalisability | **21** | Discuss the generalisability (external validity) of the study results | Discussion – Limitations (page 10): JMDC covers employer-based health insurance; self-employed, low-income, and unemployed individuals are underrepresented; external validity to the general Japanese population and other countries discussed |
| **OTHER INFORMATION** | | | |
| Funding | **22** | Give the source of funding and the role of the funders for the present study and, if applicable, for the original study on which the present article is based | Funding section (page 11): Rohto Pharmaceutical Co., Ltd. provided funding for research-related expenses including JMDC database access. Funder had no role in study design, analysis, interpretation, or publication decision |

** Give information separately for exposed and unexposed groups in cross-sectional studies, as applicable.*

*MRD = menstrual-related disorders; LEP = low-dose estrogen–progestin preparations; DNG = dienogest; ICD-10 = International Classification of Diseases, 10th Revision; CI = confidence interval; PR = prevalence ratio; PD = prevalence difference; JMDC = Japan Medical Data Center.*

*This checklist was completed in accordance with the STROBE Statement for cross-sectional studies (von Elm et al., Ann Intern Med 2007;147:573–577; https://www.strobe-statement.org).*
